# First Order Rate Law Analysis for Reset State in Vanadium Oxide Thin Film Resistive Random Access Memory Devices

**DOI:** 10.3390/nano13010198

**Published:** 2023-01-01

**Authors:** Kai-Huang Chen, Chien-Min Cheng, Na-Fu Wang, Hsiao-Wen Hung, Cheng-Ying Li, Sean Wu

**Affiliations:** 1 Department of Electronic Engineering, Center for Environmental Toxin and Emerging-Contaminant Research, Super Micro Mass Research & Technology Center, Cheng Shiu University, Chengcing Rd., Niaosong District, Kaohsiung City 83347, Taiwan; 2 Department of Electronic Engineering, Southern Taiwan University of Science and Technology, Tainan 71005, Taiwan; 3 Green Energy and Environment Research Laboratories, Lighting Energy-Saving Department, Intelligent Energy-Saving Systems Division, Industrial Technology Research Institute, Hsinchu 31040, Taiwan; 4 Department of Chemical and Materials Engineering, Lunghwa University of Science and Technology, Taoyuan 33306, Taiwan

**Keywords:** RRAM, vanadium oxide, decay, reaction rate constant

## Abstract

In the reset state, the decay reaction mechanism and bipolar switching properties of vanadium oxide thin film RRAM devices for LRS/HRS are investigated and discussed here. To discover the properties of *I-V* switching curves, the first order rate law behaviors of the reset state between the resistant variety properties and the reaction time were observed. To verify the decay reaction mechanism in the reset state, vanadium oxide thin films from RRAM devices were measured by different constant voltage sampling and exhibited the same decay reaction rate constant. Finally, the electrical conduction transfer mechanism and metallic filament forming model described by *I-V* switching properties of the RRAM devices were proven and investigated.

## 1. Introduction

Recently, non-volatile memory devices have been widely investigated because of their capacitor structure, high switching time, low operation voltage, and nonvolatile memory characteristic. Many non-volatile memory devices have recently seen wide application in modern portable electronic devices, such as PDA, cellular phone, digital camera, and flash storage. Because of the advantages of non-volatility, high operation speed, multi-state possibility, simple structure, small bit size, high packing density, and low power consumption, resistive random access memory devices (RRAM) were identified as prospective candidates for replacing traditional memory devices. Relative to the sizes of traditional electrical memory devices, such as flash memory device and dynamic random-access memory (DRAM) devices that are continually being scaled down, the memory devices reveal charge loss and reach their minimum size as limited by their physical dimensions. In addition, the various materials used in RRAM devices have been discovered and investigated in the past, such as the chalcogenide oxide materials, metal oxide-base, carbide oxide, and amorphous silicon related oxide for next-generation RRAM devices [1,2,3,4,5,6,7,8,9,10,11]. 

In this study, the Pt/V_2_O_5_/TiN structure for applications in the RRAM devices was manufactured using a Platinum electrode as the top electrode and vanadium oxide thin films, and with a TiN bottom electrode. To explain the electrical resistive switching behaviors of vanadium oxide thin film RRAM devices, a constant voltage sampling (CVS) method to measure the time-dependent *I-V* curves in the reset process was designed and discussed. In addition, the most reasonable explanation related to the initial metal filament forming process in electrochemical decay reactions for the resistive switching mechanism was also explained.

## 2. Experiment

For the structure manufacturing process of the RRAM devices, the vanadium oxide thin film was deposited by TiN/Ti/SiO_2_/Si substrate using rf-sputtering technology. The sputtering power was the rf power of 200 W for titanium nitride and dc power of 10 W for titanium targets, respectively. Next, the 200 nm thickness of the Platinum top electrode was deposited on a thin film of vanadium oxide to form RRAM devices with Pt/V_2_O_5_/TiN/SiO_2_/Si structures. The *I-V* switching characteristics of vanadium oxide thin film RRAM devices were drawn and observed by an Agilent B1500 semiconductor parameter analyzer. Additionally, the reset state relationship between the electrical properties and reaction time of vanadium oxide thin film from RRAM devices was obtained using a constant negative voltage (CVS) method. A new constant voltage sampling (CVS) method to measure the time-dependent I-V curves in reset process was designed and investigated.

## 3. Results and Discussion

Regarding to the vanadium oxide (V_2_O_5_) thin films for applications in non-volatile memory (phrase change memory, PCM) devices, two obvious resistance values were observed for V_2_O_5_ thin films at different temperatures in the heating process. The multi-level storage achieved in a phase change memory device with thin V_2_O_5_ films at fast speeds of 100 ns has been widely discussed [12].

**Figure 1:**
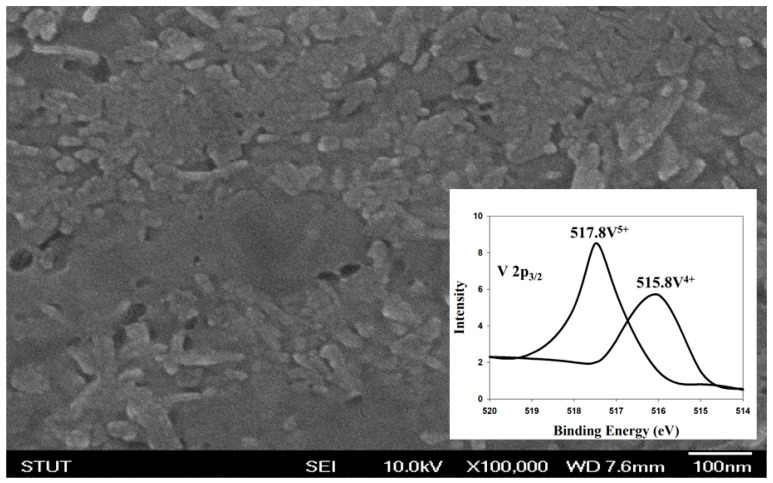
The micro-structure of the vanadium oxide thin films from the SEM surface morphology and XPS results.

**Figure 2:**
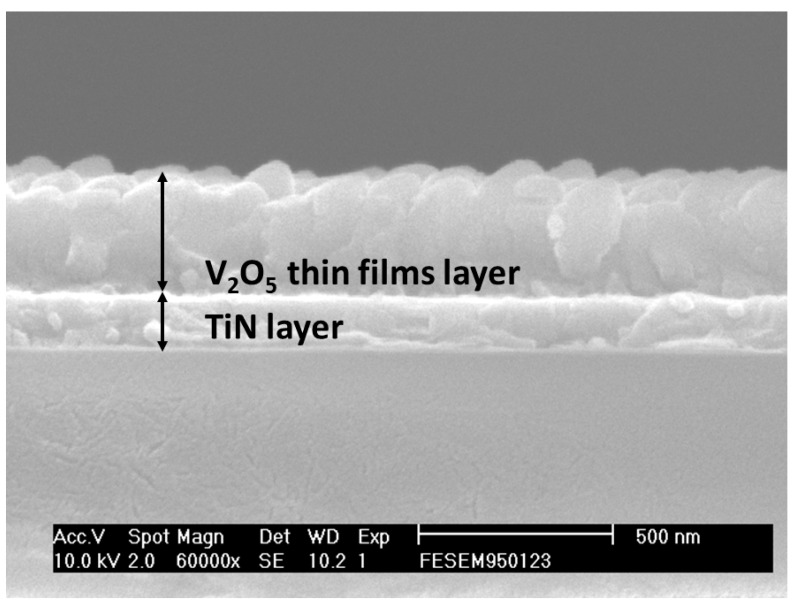
The cross-sectional images of the vanadium oxide thin films from RRAM devices.

Figure 1 shows the micro-structure of the vanadium oxide thin films of the RRAM devices from the SEM surface morphology. The specimens of thin films were about 50 nm thick and reveal dense and uniform grains. A circular plate and oval grain were also found. For XPS experimental results, the (V 2p_3/2_) dangling bonds of vanadium thin films are shown in the inset of Figure 1. The binding energy of 517.8V^4+^ and 515.8V^5+^ thin vanadium films for RRAM devices were also found. In addition, the cross-sectional images of the vanadium oxide thin films RRAM devices were observed in Figure 2. The thicknesses of the vanadium oxide thin films and TiN films of the RRAM devices were 350 nm and 200 nm, respectively.

**Figure 3:**
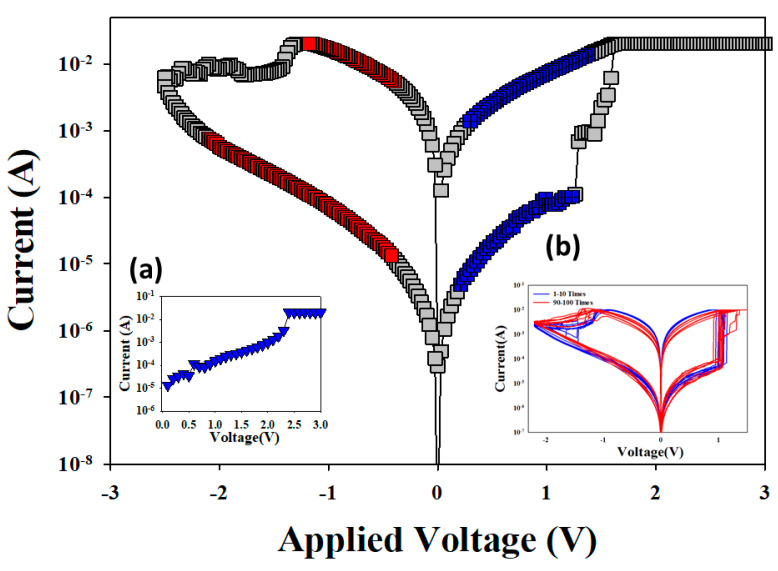
The typical *I-V* switching properties of the vanadium thin film RRAM devices for (**a**) the initial forming process, and (**b**) metal-insulator-metal (MIM) structure.

The *I–V* curves, initial forming process properties, and capacitor structure of the vanadium oxide thin film RRAM devices for the set and reset state are shown in Figure 3. The current compliance was 10 mA in Figure 3a. After the initial forming process at a voltage of 3 V, the vanadium oxide thin films from the RRAM devices reached the low resistance state (called LRS) and the high resistance state (called HRS). In order to find and confirm a stable (*I–V*) operating switching condition, the set and reset processes of the vanadium oxide thin films from the RRAM devices were repeated 100 times. The typical *I–V* curves of the RRAM devices are shown in Figure 3b, magnified by100 times.

To find and discuss the electrical conduction behavior for the metallic filament forming conduction, the Schottky emission conduction and hopping conduction mechanism were calculated by ln*I–V* and ln*I–V^1/2^* curve fitting. For the Schottky emission conduction equation,

(1) $$J=A^{*}T^{2}\exp[-q\left(\Phi_{B}-\sqrt{\frac{qE_{i}}{4\pi \varepsilon_{i}}}\right)/KT]$$

where *T* is the absolute temperature, Φ*_B_* is the Schottky barrier height, *ε_i_* is the insulator permittivity, *k* is Boltzmann’s constant, and A* is the Richardson constant. 

To calculate the $\ln(\frac{I}{T^{2}})\;$−$\sqrt{V}$ curve, the Schottky conduction mechanism equation was transformed to the *I-V* curves fitting the RRAM devices. For the hopping conduction,

(2) $$J=qN_{a}v_{0}e^{-q\Phi_{T}/kT}e^{qaV/2dkT}$$

where *d,* Φ*_T_*, *v*_0_, *N* and *a* are film thickness, barrier height of hopping, intrinsic vibration frequency, the density of space charge, and mean hopping distance, respectively [13,14,15,16]. 

**Figure 4:**
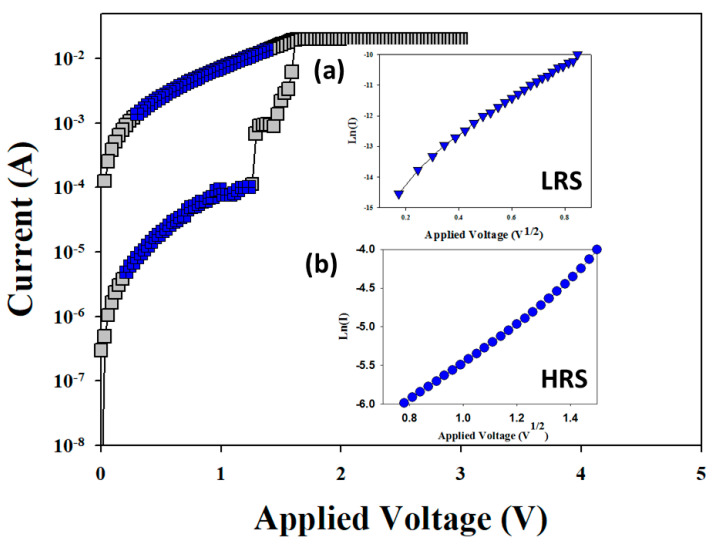
The ln(*I*)–*V*^1/2^ curve of vanadium oxide thin film RRAM devices for (**a**) LRS, and (**b**) HRS.

In Figure 4a,b, the electrical conduction mechanisms of the vanadium oxide thin films from the RRAM devices for LRS/HRS in set state were explained. In addition, different slope and intercept values of the straight-line equations of *I–V* switching curves in LRS/HRS were observed as shown in Figure 4a,b. For the LRS and HRS states, the slope value of the Schottky emission conduction in the *I–V* curves of the RRAM devices was calculated as 7.5 and 2.85, respectively. The reciprocal slope value was calculated for the Schottky emission distance value, and the intercept was calculated for the barrier height value of the Schottky conduction equation [13,14,15,16].

In Figure 4a, the electrical conduction behavior of the RRAM devices for high bias exhibited the Schottky emission mechanisms calculated from the ln*I–V*^1/2^ curve fittings. To discuss the initial metallic filament forming process applied under low voltage, the hopping conduction mechanism of the vanadium oxide thin films from the RRAM devices was calculated by ln*I–V* curve fitting. In the structure of the RRAM devices, the electron transport behavior for *I–V* curves exhibited the Schottky conduction mechanism for LRS/HRS states. In addition, the Schottky emission mechanism caused by the shallow trapped electrons jumping the activation energy barrier and inducing the lowering of the electron conduction current have been widely discussed [13,14,15,16]. 

To discuss and investigate the LRS transferred to HRS reaction mechanism for the vanadium oxide thin film RRAM devices in reset process time, the switching transition behavior and initial electrical filament forming model of the decay reaction equation in vanadium oxide thin films RRAM devices was used and defined. The equation dealing with decay rate during the electrochemical reaction is shown below,

(3) $$r=-\frac{1}{m}\frac{-d[A]}{dt}=k{\left[A\right]}^{m}{\left[B\right]}^{n}$$

where *k* is reaction constant, [*A*] is the reactant concentration, and *m/n* is the reaction order. To prove the decay trend behavior in the reset process of RRAM devices, the reaction rate relationship between reactant concentration and reaction time was transferred to ln([*A*])–*t* function for the first-order law reaction. To count the total charge quantity in *I–V* curves of RRAM devices for the reset state, Equation (4) was calculated for the reactant concentration. The reactant concentration mole [X] is equal to n×q, where *n* is the reactant valence, and *q* is one mole electron (*q* = 96,500 C). In the first order reaction equation of the decay reaction, the reaction time and the reactant concentration of the production relation in RRAM devices for reset state are dealt with.

(4) $$[\frac{\left(n\times q\right)}{V}]=\frac{mole}{V}=\left[X\right]$$

**Figure 5:**
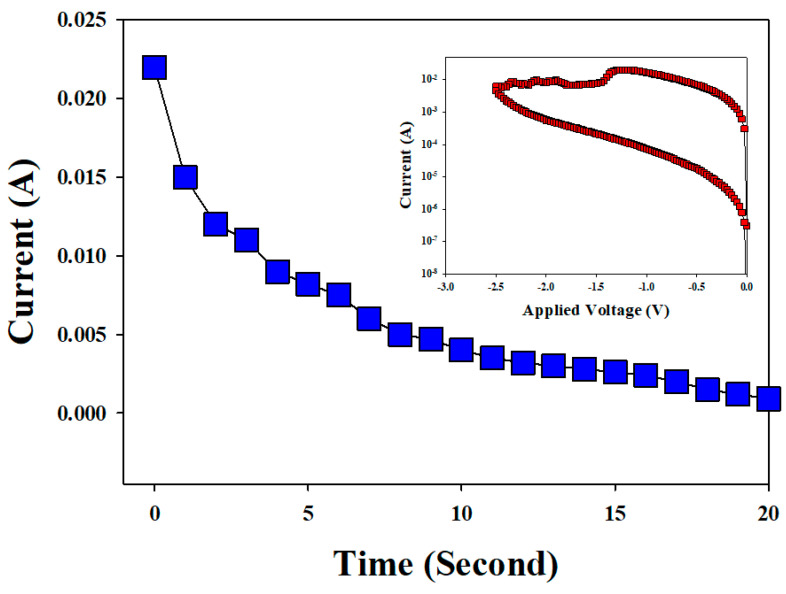
The reaction time (*S*) versus the electron charge of unit current (*A*) curves in reset state of the vanadium thin film RRAM devices for −1.5–2.5 V.

Figure 5 depicts and defines the reaction time (S) versus the current (*A*) curves in reset state of RRAM devices for −1.5~2.5 V. The total charge of the reset current (*A*) was calculated and transformed to charge (*Q*) versus reaction time curves. The total charge relationship equation between of the reaction time and the operation current is written below,

(5) Q(C)=I(A)×(t)

where the *Q* value is the instantaneous integral of the current. To effectively discuss the first order rate law for the electrochemical reaction mechanism in the reset state of the vanadium oxide thin film in the RRAM device, a CVS method was used to measure and discuss the mechanism by which a low resist state (LRS) transforms into a high resist state (HRS). Selecting a suitable negative CVS method was important to measure and confirm the resistant of the LRS state, because of its switching transition effect, long reset process time, and the heating energy effect in the reset process of the electrochemical reaction. 

**Figure 6:**
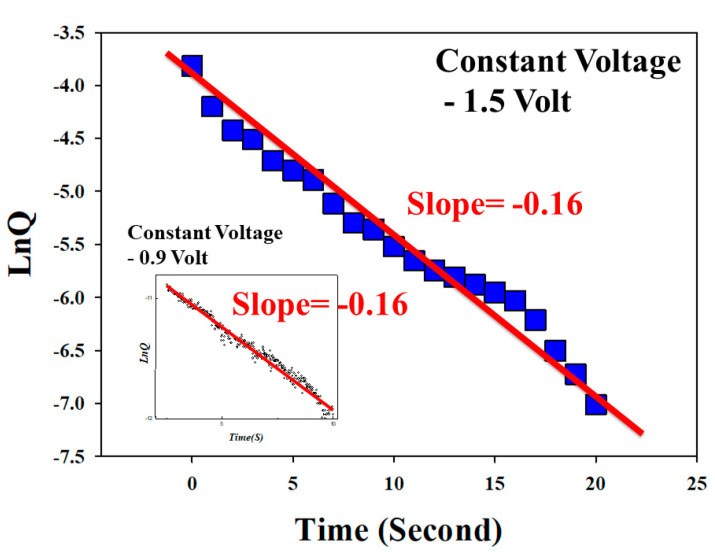
The ln*Q–T* curves of the vanadium thin film RRAM devices under −0.9 V and −1.5 V constant voltage sampling condition.

According to the equation for the linear relationship between the natural logarithm of the production concentration of reactant and reaction time, $\frac{-d\left[A\right]}{dt}=K{\left[A\right]}^{1}$, where [*A*] is the reactant concentration and k is the reaction rate constant. In addition, the mole of the reactant is equal to *Q*/(*q*×*n*), where n is the quantity of the reaction, and q is the charge of one mole electron. It means that the reactant [*A*] is proportional to the total charge number in the reset process. Because the volume of the thin film is not changed in the reset process, the equation [*Q*/(*q*×*n*)]/*V* = mole/V = [*A*] was used and measured. In Figure 6, the total charge quantity variation between the charge versus reaction time (ln*Q–T*) relationship curves of the vanadium oxide thin film RRAM devices for constant sampling voltage in the reset state was observed. The slope of the ln*Q–T* curves was transformed according to the reaction rate. According to the first order reaction law of decay, the slope of the ln*Q–T* curves in RRAM devices for −1.2 V constant sampling voltage was calculated as −0.16. In addition, the slope of the ln*Q–T* curves in RRAM devices for −0.9 V was about −0.16. From the above experimental results, the reaction rate constant in this study was found and estimated to −0.16. 

The different constant sampling voltage exhibited a similar reaction rate in all RRAM devices. The first order reaction expression equation of the decay reaction for the vanadium oxide thin film from RRAM devices in the reset state was inferred to $r=0.16{\left[O^{2-}\right]}^{1}$. 

**Figure 7:**
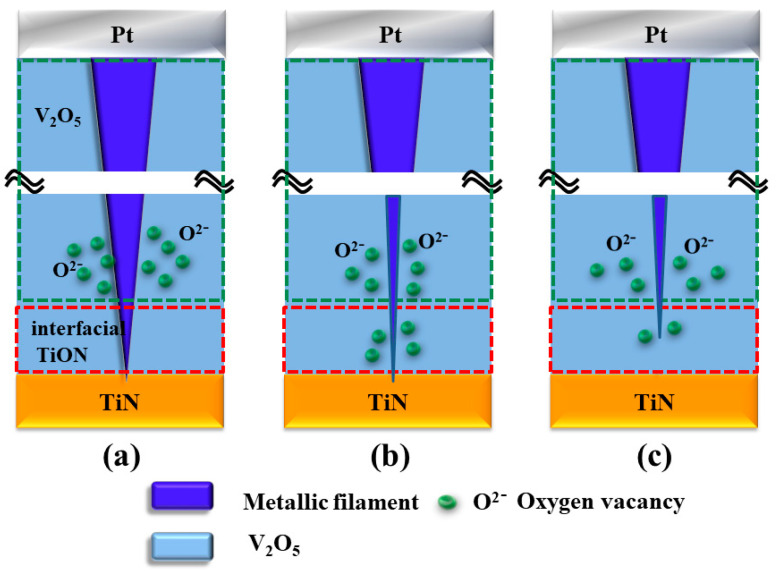
The electrical conduction model of (**a**) initial positive bias applied (**b**) the metallic filament path continuously decreased by the oxygen atoms, (**c**) the metallic filament rupture caused by oxidation reaction for the vanadium thin film RRAM devices in reset state.

Figure 7 shows and describes the electrical metallic filament-forming procedure model of the thin RRAM devices in LRS–HRS in the reset state for the electrochemical reaction. In Figure 7a, the electrical metallic filament path process for the RRAM devices for positive bias applied in the set state was described. The oxygen ions were released due to the O–O bonds being broken in the thin film layers. The vanadium metallic filament began slightly thin because of continuous oxidation behavior from oxygen atoms in the bonds of the thin V_2_O_5_ films with voltage applied. Then, other oxygen ions were released due to the O–N bond broken in the Interface between the thin film layer and the TiN electrode. In Figure 7b, the vanadium metallic filament was continuously decreased in size by the oxygen atoms in the TiN interface layer. In Figure 7c, the released oxygen ions were driven by the electric field to the conductive metallic filament, leading to the rupture of the filament due to an oxidation reaction. Finally, the resistance state of the thin film from RRAM devices switched from LRS to HRS to complete the reset process [17,18,19,20,21,22,23].

**Figure 8:**
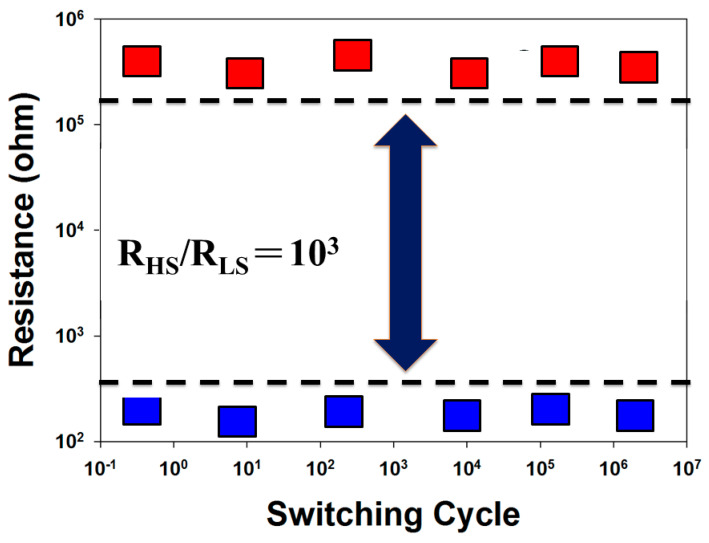
The resistance value versus time curves of the vanadium thin film RRAM devices.

**Figure 9:**
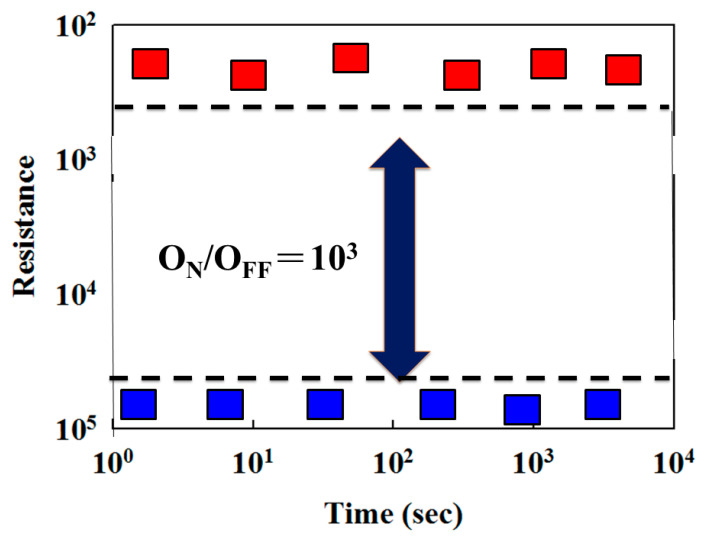
The resistance value versus switching cycle curves of the vanadium thin film RRAM devices.

The switching cycling testing measurement was carried out using the type of the retention properties and endurance test characteristics. In Figure 8, the retention characteristics of the thin film RRAM devices for the LRS/HRS resistance states were measured to investigate the reliability for applications in non-volatile memory RRAM devices. In Figure 9, no significant changes in the switching resistance cycling versus time curves in thin film RRAM devices for the LRS/HRS states were found for more than 104 s from the extrapolation calculation anticipation.

Finally, we found and confirmed that the LRS–HRS reaction in the I-V curves of the vanadium oxide thin films RRAM devices for the reset process was related to the reaction rate constant of the first order chemical reaction in the electrochemical reaction in this study. 

## 4. Conclusions

In conclusion, the bipolar switching properties for the decay trend in the reset status of vanadium thin film RRAM devices were discussed and investigated. In addition, constant voltage sampling was used to measure and define the LRS/HRS transformation conduction mechanism of the vanadium thin film RRAM devices in the reset state. For the ln*Q-T* curves, the reaction rate constant of the first order reaction law of the vanadium thin film RRAM devices for the different constant voltage sampling was shown to be −0.16. Finally, the first order decay reaction expression equation of vanadium thin film RRAM devices in LRS/HRS transformed reaction for reset state was proven to be $r=0.16{\left[O^{2-}\right]}^{1}$.
